# Single subject pharmacological-MRI (phMRI) study: Modulation of brain activity of psoriatic arthritis pain by cyclooxygenase-2 inhibitor

**DOI:** 10.1186/1744-8069-1-32

**Published:** 2005-11-02

**Authors:** M Baliki, J Katz, DR Chialvo, AV Apkarian

**Affiliations:** 1 Departments of Physiology, Northwestern University Feinberg School of Medicine, Chicago, Illinois, 60611, USA; 2 Anesthesiology, Northwestern University Feinberg School of Medicine, Chicago, Illinois, 60611, USA

## Abstract

We use fMRI to examine brain activity for pain elicited by palpating joints in a single patient suffering from psoriatic arthritis. Changes in these responses are documented when the patient ingested a single dose of a selective cyclooxygenase-2 inhibitor (COX-2*i*). We show that mechanical stimulation of the painful joints exhibited a cortical activity pattern similar to that reported for acute pain, with activity primarily localized to the thalamus, insular, primary and secondary somatosensory cortices and the mid anterior cingulum. COX-2*i *resulted in significant decreased in reported pain intensity and in brain activity after 1 hour of administration. The anterior insula and SII correlated with pain intensity, however no central activation site for the drug was detected. We demonstrate the similarity of the activation pattern for palpating painful joints to brain activity in normal subjects in response to thermal painful stimuli, by performing a spatial conjunction analysis between these maps, where overlap is observed in the insula, thalamus, secondary somatosensory cortex, and anterior cingulate. The results demonstrate that one can study effects of pharmacological manipulations in a single subject where the brain activity for a clinical condition is delineated and its modulation by COX-2*i *demonstrated. This approach may have diagnostic and prognostic utility.

## Introduction

Over the last fifteen years, functional MRI (fMRI) and positron emission tomography (PET) have been used to unravel brain circuitry underlying pain perception and study the properties of these areas in acute and chronic pain conditions (for a recent review see [1]). Recently, the utility of combining fMRI with pharmacology has been demonstrated by a number of groups [2-6]. Brain imaging studies in combination with various analgesics have also been described regarding the impact of examined chemicals on brain activity for pain [7-11]. These studies examine acute experimental pain conditions, and demonstrate the possibility of delineating brain regions modulated in normal subjects, for centrally acting drugs, such as opiates and ketamine. In the present study we show the potential of studying clinical pain conditions, and tracking the efficacy of pharmacological interventions in an individual patient, where multiple repeat scans are performed before and after administering a single dose of the analgesic that the patient was using to manage satisfactorily his arthritis.

We examine the analgesic efficacy of a selective cyclooxygenase-2 (COX-2) inhibitor on psoriatic arthritis pain. Traditional nonsteroidal anti-inflammatory drugs (NSAIDs) are nonselective inhibitors of COX-1 and COX-2, which catalyze transformation of arachadonic acid to prostaglandin. Substantial clinical evidence shows that COX-2 selective inhibitors are effective for treating osteoarthritis, rheumatoid arthritis, and other inflammatory pain conditions [12]. Differential elevation of COX-2 has been documented in synovial tissue of patients with inflammatory arthritis, including patients with psoriatic arthritis [13], and in animal studies of inflammatory pain COX-2 elevation is observed in the periphery and in the central nervous system [14]. Thus, there is good scientific evidence for management of inflammation and pain of psoriatic arthritis with selective COX-2 inhibitors (COX-2*i*). Here we examine the effects of a single dose of a COX-2*i *on brain activity for joint pressure allodynia associated with psoriatic pain.

## Results

A single subject with psoriatic pain was studied. The subject skipped a single dose of his COX-2i 12 hours prior to the scanning session. Brain activity was performed for palpating the painful joints in 4 fMRI scans prior to administration of COX-2i, in 4 fMRI scans 1 hour post drug ingestion, and in 2 fMRI scans 3 hours post drug ingestion.

### COX-2*i *treatment decreased pain

**Figure 1:**
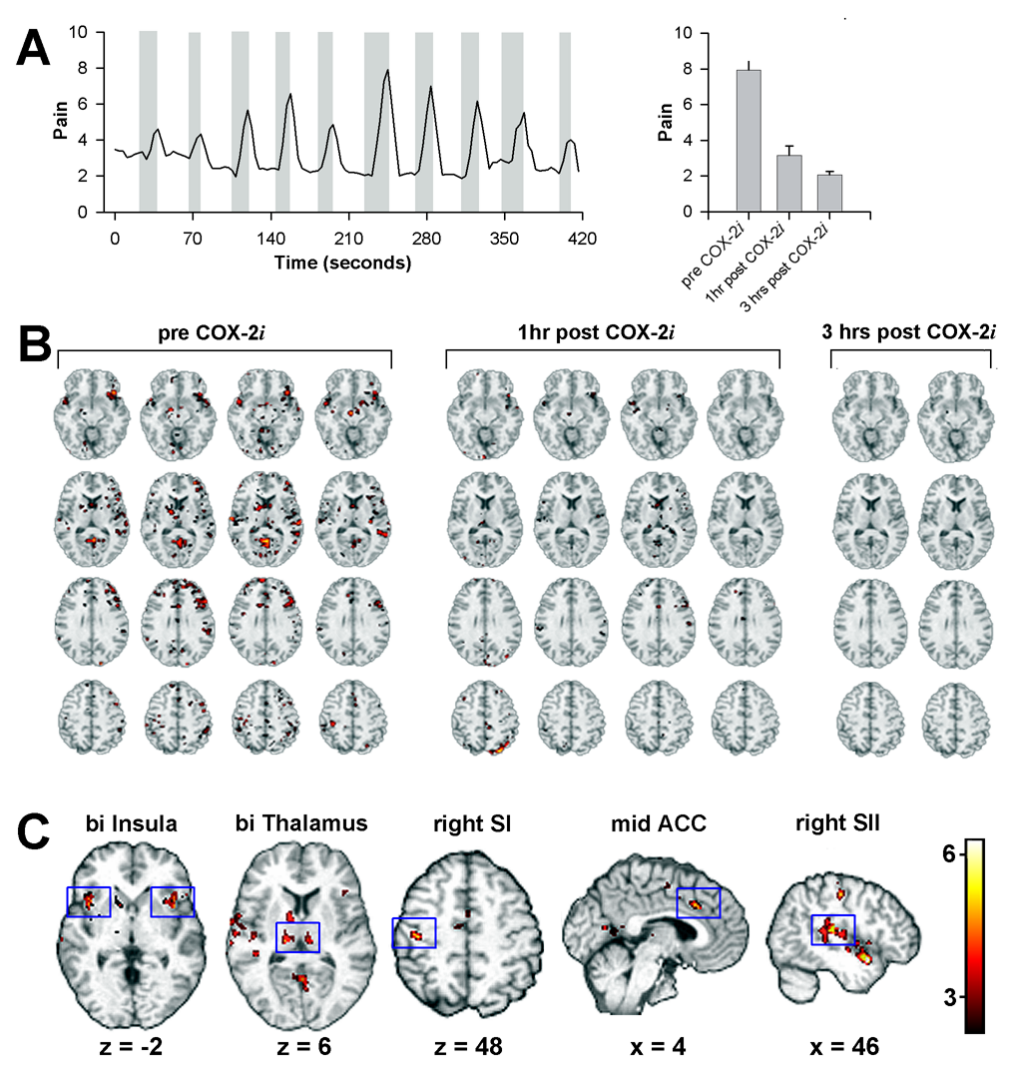
**A**. Example pain intensity rating for manually palpating painful arthritic joints (grey areas is duration of palpation). Right panel shows that the mean rating of pain intensity for palpating painful joints, which significantly decreased 1 and 3 hours after administration of COX-2i when compared to the baseline (p < 0.001). **B**. Brain activity in ten consecutive scans is presented for palpating painful joints (each column is a separate scan). The first 4 scans are prior to drug ingestion (pre COX-2i), the next four start 1 hour post drug ingestion, the last two are 3 hours post-drug ingestion. Four transverse slices are shown for each scan (top to bottom: z = -12, 10, 28 and 44). Note that brain activity decrease after cox-2i is evident in these individual scans. **C**. Average group activity minus visual controls using fixed effects analysis for palpating painful joints across all conditions (pre and post COX-2*i*). Activity is seen bilaterally in insular cortex and thalamus, in addition to right primary and secondary somatosensory areas and mid anterior cingulum. Activity maps are presented in MNI space, x y z coordinates in mm.

A single 200 mg dose of selective COX-2i reduced baseline pain, joint stimulation pain, and restored ability to ambulate. At the start of the study the patient was not able to stand on his legs due to severe ankle joint pain. One hour after ingesting the medicine he still had very limited mobility. After three hours, he was able to walk. Left panel of Figure 1A shows an example rating of pain when joints of the hand are palpated (collected during an fMRI session, prior to ingesting the COX-2i), where the average baseline pain is about 3 and stimulus-evoked pain is about 6 (on a 0–10 pain scale, 10 = maximum imaginable pain). The medication decreased baseline pain by 50% (over 4 hours post-treatment); stimulus evoked pain from 7.93 +/- 0.73 to 3.15 +/- 0.55 (1 hour post COX-2*i*, p < 0.001), to 2.2 +/- 0.12 (3 hours post COX-2*i*, p < 0.001 (right panel of figure 1A).

### COX-2*i *treatment decreased pain related brain activity

We observe widespread brain activity for palpating painful joints prior to drug administration. This activity was significantly attenuated across all areas 1 hour after ingestion of COX-2*i*, and there was no significant brain activity after 3 hours of drug ingestion (figure 1B).

Overall brain activity for palpating painful joints was determined by averaging all 10 scans, prior and post-drug ingestion. The activity mainly included areas coding the sensory properties of the acute painful stimulus, including bilateral anterior and posterior insula together with secondary somatosensory cortex, multiple portions of anterior cingulate, primary somatosensory cortex, basal ganglia, and thalamus (Figure 1C, Table I).

**Table 1:** Brain regions activated for palpating painful joints in a psoriatic arthritis patient

**Region**	**Coordinates**			**Z value**	**Cluster Index**	**Voxels**	**P value**
	**x**	**y**	**z**				
R sup temporal pole	46	6	-6	6.24	10	835	p < 10^-14^
R ant insula *	40	20	-6	3.98	10		
R inf parietal, S2 *	42	-22	16	3.93	10		
L ant insula *	-42	22	-2	4.66	9	321	p < 10^-06^
L ant insula/inf frontal (45/47)	-48	20	-6	4.52	9		
R ant thalamus	10	-4	6	3.65	8	258	p < 10^-05^
R putamen	26	6	-4	2.70	8		
R S1 hand (3) *	44	-20	48	5.43	7	210	p < 10^-04^
R S1/M1 hand (3/4)	38	-28	68	4.08	7		
R mid temporal	68	-22	8	4.56	6	203	0.000117
bi lingual	-4	-66	10	4.57	5	200	0.000136
R thalamus *	16	-22	8	4.56	4	187	0.000263
mid ACC (24) *	2	22	34	4.48	3	158	0.00122
R SMA (6)	10	-6	52	3.68	2	135	0.00439
L thalamus *	-12	-24	6	4.01	1	123	0.0088

L = left; R = right; bi = bilateral; mid = middle; ant = anterior; inf = inferior; post = posterior; sup = superior ; S1 = primary somatosensory cortex; S2 = secondary somatosensory cortex; M1 = primary motor cortex; ACC = anterior cingulate; SMA = supplementary motor area; numbers in parenthesis = Brodmann areas. Activity for brain areas marked by * are shown in Figure 1C.

**Figure 2:**
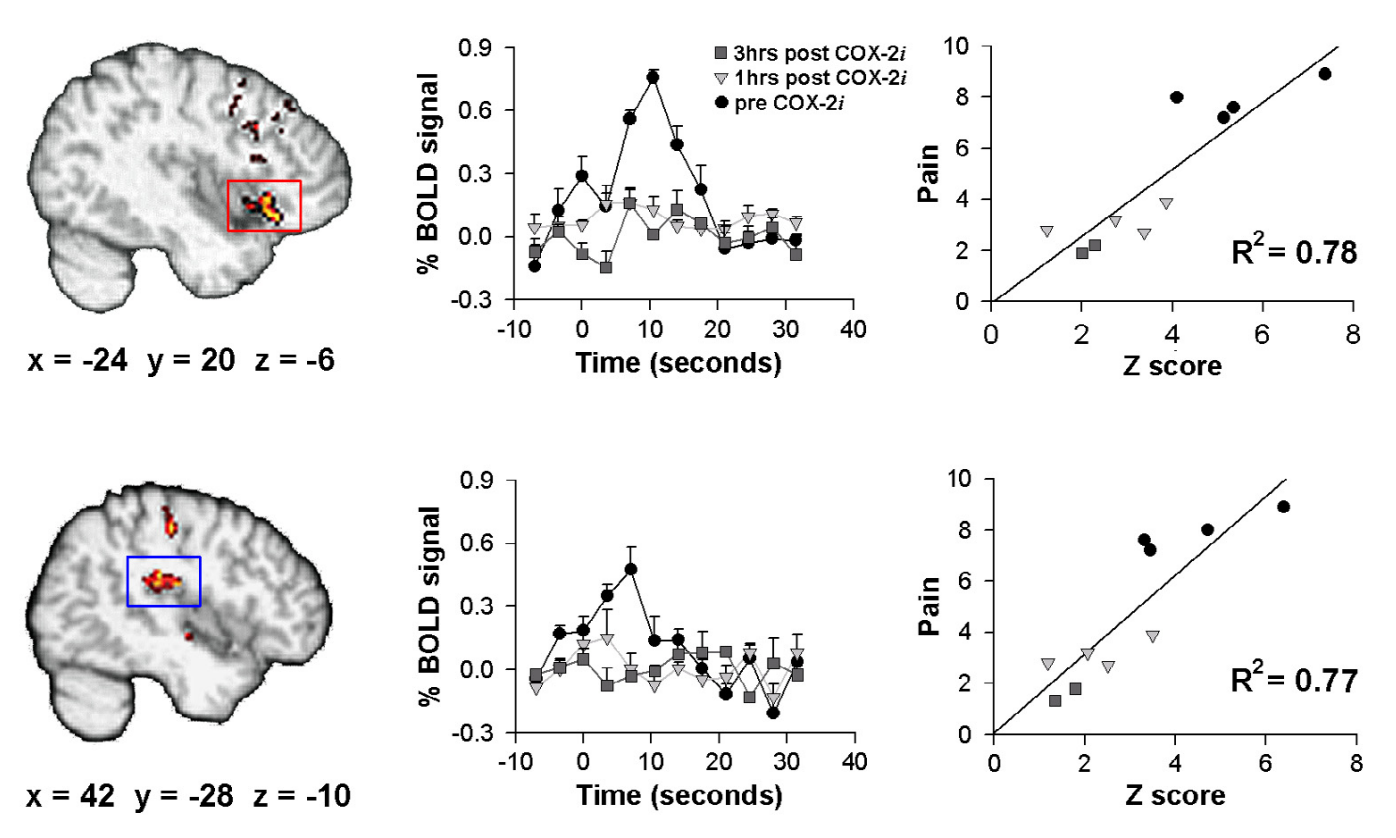
Higher-level covariate analysis between pain intensity for palpating joints (mean rating of pain during scan) and brain activity across all 10 scans results in two main clusters: left insular cortex (red) R^2 ^= 0.78 and right secondary somatosensory area (blue) R^2 ^= 0.77. Middle panels show the average change in BOLD signal during stimulation (mean stimulus duration = 13.3 seconds) for these areas prior to, and 1 and 3 hours after the administration of COX-2*i*. Right panels show the relationship between activity (Z score) and pain for a 1 cc volume centered at the peak of the covariate analysis, for each region.

Brain areas that correlated with pain intensity ratings are shown in figure 2. The main areas include anterior insular cortex, and secondary sensory/posterior insular cortex. The average BOLD signal in these two areas shows the response profile of brain activity in each region and the decrease in magnitude of this response after drug ingestion. In both regions, magnitude of activity (Z-values) is strongly correlated with pain ratings (figure 2 last two panels).

It is worthy to note that no brain areas showed significant increases in activity after the administration of drug (i.e. the contrast: post – pre drug ingestion scans yielded no positive results, and no brain areas were negatively correlated with pain). Thus, we could not identify any brain area responding to the drug.

### Overlap between brain activity in the patient with psoriatic pain and acute thermal pain in normal subjects

**Figure 3:**
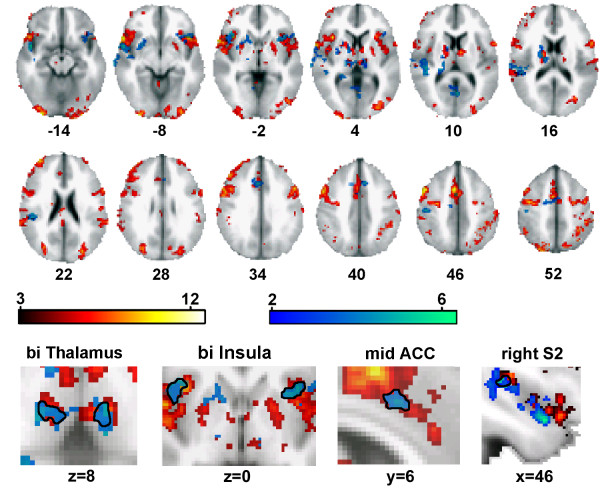
Similarity of brain activity for psoriatic pain in a single subject to thermal pain in a group of normal subjects. Top panel: The close proximity of brain activity is shown for the entire brain. Average group activity maps for thermal painful stimuli in 7 healthy control subjects (red) and for palpating painful joints in one patient (blue). Conjunction analysis between heat pain in healthy normal subjects and psoriatic arthritis pain indicates regions of overlap (outlined in black in lower panels), including: bilateral insula (z = -8 to +4) and thalamus (z = +4), ACC (z = 34), and right S2 region (z = +10 to +22\).

The average brain activity pattern for palpating joints in the psoriatic arthritis patient closely resembles brain activity observed for acute pain, as reported by many groups in the past, see recent review [1]. To directly demonstrate this similarity we compared the spatial activity pattern in this patient to group-averaged brain activity in normal subjects following thermal painful stimulation. This similarity is illustrated in figure 3. The overlap between these activation maps included bilateral thalamus and insula, anterior cingulate, and secondary somatosensory cortex.

## Discussion

The study demonstrates feasibility of studying effects of a single dose of an analgesic on brain activity for a clinical pain condition in an individual subject. We show that even in a single subject using repeated fMRI scans it is possible to demonstrate the efficacy of an analgesic in relation to brain activity, to identify the regions involved in the pain, and the regions specifically modulated by the analgesic.

Peripheral vs. central actions of COX-2 inhibitors remains unknown in man, although there is evidence for their central efficacy in rodents [14]. We could not identify brain regions responding to the drug, independent of pain modulation, implying that the drug-induced analgesia is probably not mediated through active inhibition at the level of the cortex. However, this negative finding may also be due to many other reasons (like insufficient power), especially since this is a single subject study, and needs to be determined in a larger population of patients.

The pattern of brain activity observed for the psoriatic arthritis joint pain is similar to brain activity observed by many groups for acute pain, and especially for acute thermal painful stimuli, see for example [15-18], and many others [1]. A recent meta-analysis of human brain imaging studies regarding regional activity for acute pain indicates that incidence of activity for anterior cingulate, primary somatosensory, secondary somatosensory, and insular cortices, as well as the thalamus ranges from 75% to 94% [1]. Consistent with these observations recent rodent studies indicate that manipulating activity in anterior cingulate and insula can modify pain-like behavior [19-21]. All of these regions were identified as activated in our psoriatic arthritis patient, and most of them (all but primary somatosensory cortex) showed overlap of activity between psoriatic pain and thermal pain in normal subjects. Anterior insula and secondary somatosensory cortex/posterior insula were the two brain regions that robustly reflected changes in pain perception as a consequence of ingesting the COX-2*i*. A number of groups have studied brain regions specifically coding painful stimuli for acute pain [16,22,23]. The study by Bornhovd et al. [22] examined laser stimulation evoked responses and differentiated between detection of painful stimuli vs. coding of painful stimuli, and showed that anterior insula and secondary somatosensory cortex are the ones that best code pain intensity. Thus, the psoriatic pain modulation by COX-2*i *closely agrees with the results of Bornhovd et al. [22]. In contrast, our recent study of brain activity in chronic back pain patients with radiculopathy, and using a data collection and analysis approach identical to the present study, indicated that back pain intensity was represented in medial prefrontal cortex activity, while duration of back pain was reflected by anterior insula activity (preliminary report by Baliki et al. Society Neuroscience Abstract 2003). Therefore, at least in the subject that we have studied, the psoriatic arthritis joint pain seems more like acute thermal pain and less like chronic neuropathic back pain. This result is consistent with the clinical assessment of psoriatic arthritis being more an inflammatory condition than neuropathic [13] and the patient's responsiveness to the COX-2*i *is in agreement with animal studies showing that COX-2 expression and responsiveness to COX-2*i *seems specific to inflammatory rather than neuropathic pain conditions [14,24], but of course this notion remains to be demonstrated in a population of psoriatic arthritis patients.

In conclusion, this case study has important clinical implications in making judgments as to the efficacy of pharmacotherapies on brain circuitry for clinical pain conditions that a patient may be suffering from, and demonstrates a method that can identify brain regions that may differentiate between patients with similar clinical conditions but with different responses to the same pharmacotherapy. In general, the methodology provides an objective approach that may be used for drug development and testing effects of drugs in individual cases.

## Materials and methods

A single patient with psoriatic arthritis pain in multiple joints was recruited to the study. An additional 7 healthy subjects were studied for thermal pain. Subjects were instructed regarding the procedure and signed a consent form. Institutional Review Board of Northwestern University approved the procedures.

The patient agreed to skip one dose of the analgesic and anti-inflammatory selective COX-2i (200 mg Celebrex every 12 hours, Pfizer) 12 hours prior to the start of the study. The subject knew that this would dramatically limit his mobility and requested help in transportation from home to the scanner. The subject rated his pain on a verbal analog scale (0 = no pain, 10 = most imaginable pain) at the start of the study and at different time intervals throughout the course of the study. The participant's ability to ambulate was tested at the start and at various intervals during the study, as an alternate measure for knee joint pain. He underwent 10 joint palpation scans, 4 prior and 6 after ingesting the analgesic, 2 fMRI scans for visual-motor controls, and anatomic scans for proper registration of the fMRI scans to standard space.

### Brief outline of case history

The subject was a 53 year old male with a past medical history significant for plantar-palmar psoriasis and mild gastric reflux from a small hiatal hernia. Past medical history was notable only for a lack of any orthopedic procedures on any joints. The psoriasis had been present since age 39, but 7 months prior to his participation in this investigation, the psoriasis became acutely more severe but remained purely a dermatologic problem. At 3 months prior to the investigation, the patient noted an acute onset of swelling in the left knee associated with significant pain on movement. This was clinically diagnosed as psoriatic arthritis, and within weeks involved all joints of the extremities with severe swelling and pain with movement. Further, pain along the entire spine was noted. The severity of the pain significantly interfered with sleep and with all activities of daily living. Daily doses of ibuprofen of 3.2 g and of naprosyn 2.4 g failed to control the pain. After 3 months of poorly controlled pain, he was started on celecoxib 200 mg bid. Within 1 hour of the first dose, profound relief was noted, and this relief was sustained throughout the use of the medication allowing him to sleep and restore full activities of daily living. However, despite the pain control with the celecoxib, joint swelling was unchanged.

### Brain Imaging

Brain activity was studied with fMRI while the patient rated fluctuations of ongoing pain using a finger-spanning device to continuously rate and log subjective perception of pain during fMRI data collection [25]. The patient underwent an initial training phase prior to scanning, in which he learned to use the finger-span device. During brain imaging sessions, the finger span device is synchronized and time locked with fMRI image acquisition and connected to a computer providing visual feedback. This device was also used in performing visual control scans.

### Rating pain during stimulation of arthritic joints

The patient rated the stimulus pain with the finger-span device. During a given functional imaging session, 10 stimuli ranging in duration from 10 to 15 seconds were applied to the arthritic limbs and digits. (see fig 1A). The patient rated pain on a scale of 0 to 10 and was provided with a visual feedback of the rating. The stimulus was delivered manually by an investigator who applied variable duration and intensity pressures on the painful joints. Stimulus intensity was non-painful when applied on the investigator but was always rated painful (of variable intensity) by the patient. In some scans stimuli were applied to the hand joints (left and right), in others to the kneecap (left and right). Given the small number of fMRI scans, we did not attempt to distinguish between stimulus sites (neither body side nor upper vs. lower limb distinctions were tested).

### Rating pain during thermal stimulation in healthy normals

Healthy volunteers (n = 7; average age = 30.3 ± 3.2) were scanned during acute thermal stimulation where they rated the stimulus pain with the finger-span device. During a given functional imaging session, 9 noxious thermal stimuli ranging in duration from 10 to 30 seconds were applied to the healthy subjects. The stimuli were applied both on the lower back area and volar surface of the hands. Subjects were instructed to rate their pain on a scale of 0 to 10 and were provided with a visual feedback of their rating (average reported pain across all subjects and runs was 5.0 ± 1.6). A purpose built, fMRI compatible thermal stimulator delivered fast ramping (10°C per second) painful thermal stimuli (baseline 38°C, peak temperatures 46°C and 48°C) via a contact probe (1 × 1.5 cm peltier). Durations and intensities of thermal stimuli as well as inter-stimulus intervals were presented in a pseudorandom fashion. This variation in interval was adopted to decrease the regularity of stimulus presentation and thus reduce volunteers' ability to predict the arrival of the next stimulus. In addition, the relatively long duration inter-stimulus interval reduced sensitization.

### Visual control

The patient and normal subjects were instructed to follow as closely as possible fluctuations of a bar projected on a screen in time. This visual tracking provides an adequate visual-motor control since it is similar to the pain rating finger-span task, with the important difference being that now the finger movement (i.e. variations in magnitude) is correlated with a visual input rather than pain.

### Functional Magnetic Resonance Data Analysis

Functional MR data for the arthritic patient was acquired with a 1.5T Siemens (VISION) whole-body scanner with echo-planar imaging (EPI) capability using the standard radio-frequency head coil. Multi-slice T2*-weighted echo-planar images were obtained with the following parameters: repetition time TR = 3.5 s, echo time TE = 70 ms, flip angle = 90°, slice thickness = 3 mm, in-plane resolution = 3.475 × 3.475 mm^2^. The 36 slices covered the whole brain from the cerebellum through to the vertex. An average of 240 volumes were acquired per event per condition in all participants. A No-Flow T1-weighted anatomical MRI image was also using the following parameters: TR = 22 ms, TE = 5.6 ms, flip angle = 20°, matrix 256 × 256 and a FOV of 240 mm, with 160 mm coverage in the slice direction. Anatomic images were used to register functional images in standard space.

Functional MR data for thermal pain in normal healthy subjects were acquired with a 3T Siemens Trio whole-body scanner with echo-planar imaging (EPI) capability using the standard radio-frequency head coil. Multi-slice T2*-weighted echo-planar images were obtained with the following parameters: repetition time TR = 2.5 s, echo time TE = 70 ms, flip angle = 90°, slice thickness = 3 mm, in-plane resolution = 3.475 × 3.475 mm^2^. The 36 slices covered the whole brain from the cerebellum to the vertex. An average of 400 volumes were acquired per event per condition in all participants. A T1-weighted anatomical MRI image was also acquired for each subject using the following parameters: TR = 2.1 s, TE = 4.38 ms, flip angle = 8°, FOV = 220 mm, slice thickness = 1 mm, in-plane resolution = 0.86 × 0.86 mm^2 ^and number of sagittal slices = 160.

Image analysis to reveal significant brain activity based on changes in BOLD signal was performed using FMRIB Expert Analysis Tool (FEAT, [26], ). The preprocessing of each subject's individual scan time-series of fMRI volumes encompassed: slice time correction; motion correction using MCFLIRT; spatial smoothing using a Gaussian kernel of full-width-half-maximum 5 mm; nonlinear high-pass temporal filtering (Gaussian-weighted least-squares straight line fitting, filter cutoff of 100 seconds) and subtraction of the mean of each voxel time-course from that time-course (i.e. intensity normalization). The fMRI signal was then linearly modeled on a voxel by voxel basis using FMRIB's Improved Linear Model (FILM) with local autocorrelation correction [27,28].

Each condition described above (thermal pain, arthritic pain and visual ratings) was considered to generate a hemodynamic response described by the convolution of the corresponding finger span rating with a generalized hemodynamic response function (gamma function: lag = 6 seconds, standard deviation = 3 seconds). Head motion vector (derived from motion correction) was used at this level as a covariate of no interest to further remove any residual variance due to head motion. The significance of the model fit to each voxel time-series was calculated, yielding statistical parametric maps for each scan. Average group activity maps were generated by subtracting the visual control scans from the pain activity maps using FEAT in a second level random and fixed effects group analysis, following the co-registration of individual scans to standard space (152 subject average Montreal Neurological Institute (MNI) space, ). This results in a *Z*-score map of statistically significant pain-related activity across different conditions. For random-effects, cluster-based correction of the *Z*-statistic images was performed. The raw *Z*-statistic images from the group analysis were thresholded at *Z*-scores > 2.3. For each resulting cluster of spatially connected voxels surviving the *Z *threshold, a cluster probability threshold of *P *= 0.01 was applied to the computed significance of that cluster, which corrects for multiple comparisons [29].

Stereotactic coordinates of local maxima within areas of significant activity change were determined for each condition. The localization of these local maxima and clusters was determined and assessed by reference to a standard stereotactic atlas [30] and reported in MNI coordinates.

Covariate analysis for arthritic pain was performed for all stimulation scans with mean pain rating of each scan. This is a higher level analysis where first level brain activity maps are used to determine brain regions that correlate across all scans with the corresponding pain intensity. The resultant map shows clusters of voxels that significantly covary with pain intensity across scans. Based on the results of this analysis, we perform a regional correlation analysis using the mean Z-value of a 27-voxel (1 cc) volume centered at the peak within a cluster of interest, relating this value to pain intensity across scans.

Central activation by COX 2i was investigated by a higher level analyses that was performed to determine brain areas that shows increased activity post COX-2i. A simple linear model was utilized in the set up of this covariate analyses: scans performed 3 hours post, 1 hour post and pre COX-2i were regressed with 1,0,-1 respectively. No brain areas exhibited significant corrolation (i.e. no voxels had a linear increase in activity across the 3 sessions).

Average time-course of BOLD response for arthritic pain was calculated for each fMRI scan by and then averaged across session. Pre-processed fMRI images are re-averaged relative to the start time of stimuli, 2 brain volumes (7 seconds) prior to the start time and 9 (31.5 seconds) post-start time. The average duration of stimuli across all scans is 13.3 seconds. Time-courses for coordinates of interest are then extracted from each scan, averaged over the 27-voxel neighborhood, and then averaged across sessions. Variability of these curves is expressed in S.E.M. over the scans. These BOLD responses show the time-course of activity in a given brain region relative to the stimulus, and indicate its changes at different time points from drug ingestion.

The thermal pain responses in normal subjects was used to indicate the spatial similarity between this map and the map obtained for psoriatic pain. Overlap between the two maps was identified using conjunction analysis, where a spatial mask is generated from the group-averaged thermal map, thresholded as Z-value > 2.3. The intersection between this mask and brain activity for psoriatic pain (averaged across all scans) determines brain regions commonly activated in both conditions. It is important to note that no statistical comparisons were made for intensity of activity between maps for thermal stimulation in normal healthy volunteers and arthritic pain since each data were collected on different strength magnets.
